# C-Terminal Alpha-1 Antitrypsin Peptide: A New Sepsis Biomarker with Immunomodulatory Function

**DOI:** 10.1155/2016/6129437

**Published:** 2016-06-13

**Authors:** Nancy Blaurock, Diana Schmerler, Kerstin Hünniger, Oliver Kurzai, Katrin Ludewig, Michael Baier, Frank Martin Brunkhorst, Diana Imhof, Michael Kiehntopf

**Affiliations:** ^1^Department of Clinical Chemistry and Laboratory Medicine, Jena University Hospital, Erlanger Allee 101, 07747 Jena, Germany; ^2^Septomics Research Center, Friedrich Schiller University and Leibniz Institute for Natural Product Research and Infection Biology, Hans-Knöll-Institute (HKI), Albert-Einstein-Street 10, 07745 Jena, Germany; ^3^Center for Sepsis Control and Care (CSCC), Jena University Hospital, Erlanger Allee 101, 07747 Jena, Germany; ^4^Department of Anesthesiology and Intensive Care, Jena University Hospital, Erlanger Allee 101, 07747 Jena, Germany; ^5^Department for Medical Microbiology, Jena University Hospital, Erlanger Allee 101, 07747 Jena, Germany; ^6^Paul-Martini-Research Group, Jena University Hospital, Erlanger Allee 101, 07747 Jena, Germany; ^7^Pharmaceutical Institute, Pharmaceutical Chemistry I, University of Bonn, Brühler Street 7, 53119 Bonn, Germany

## Abstract

Systemic inflammatory response syndrome (SIRS) is a life threatening condition and the leading cause of death in intensive care units. Although single aspects of pathophysiology have been described in detail, numerous unknown mediators contribute to the progression of this complex disease. The aim of this study was to elucidate the pathophysiological role of CAAP48, a C-terminal alpha-1 antitrypsin fragment, that we found to be elevated in septic patients and to apply this peptide as diagnostic marker for infectious and noninfectious etiologies of SIRS. Incubation of human polymorphonuclear neutrophils with synthetic CAAP48, the SNP-variant CAAP47, and several control peptides revealed intense neutrophil activation, induction of neutrophil chemotaxis, reduction of neutrophil viability, and release of cytokines. We determined the abundance of CAAP48 in patients with severe sepsis, severe SIRS of noninfectious origin, and viral infection. CAAP48 levels were 3-4-fold higher in patients with sepsis compared to SIRS of noninfectious origin and allowed discrimination of those patients with high sensitivity and specificity. Our results suggest that CAAP48 is a promising discriminatory sepsis biomarker with immunomodulatory functions, particularly on human neutrophils, supporting its important role in the host response and pathophysiology of sepsis.

## 1. Introduction

The acute phase protein alpha-1 antitrypsin (AAT) is a circulating protease inhibitor belonging to the serpin superfamily with divergent immunomodulatory functions, such as reduced production of proinflammatory cytokines [1–3], inhibition of neutrophil activation and chemotaxis [4–6], and suppressed apoptosis of hepatocytes or vascular smooth muscle cells [7, 8]. However, administration of high dose AAT in a primate sepsis model exacerbated septic shock, mainly due to high concentrations of cleaved AAT, which has been shown to induce a strong immune reaction [9].

Cleavage of AAT occurs at the reactive center loop (RCL) by target proteases (e.g., neutrophil elastase) as well as nontarget proteases (e.g., matrix metalloproteases) [10]. Both reactions lead to a conformational change and a loss of inhibitory activity. Cleavage between Phe^352^-Leu^353^ generates a carboxy-terminal 42-residue peptide with a mass of 4789 Da (CAAP48), which is bound to the cleaved complex in a noncovalent manner [11, 12]. Variants of this fragment such as VIRIP or C-36 peptide have been identified in several human tissues [13–15] and body fluids [16–18]. For these C-terminal fragments physiological functions, such as NK-cell suppression and serine protease protection (CRISPP peptide [19–21]), extracellular matrix protection by reversible serine protease inhibition (SPAAT [14]), pro- or anti-inflammatory immune modulating functions (C-36 peptide [1, 15, 22]), and inhibition of HIV entry (VIRIP [18, 23]) have been reported.

In the AAT gene over 75 polymorphisms have been described [24]. One frequent SNP (rs1303, MAF = 0.28), leading to an E > D substitution, occurs in the sequence of CAAP48. Thus cleavage of AAT between Phe^352^ and Leu^353^ in the presence of SNP rs1303 leads to the generation of a cleavage product with a lower molecular mass of 4775 Da, designated CAAP47. So far the* in vivo* relevance of this polymorphism is unclear.

Sepsis is a leading cause of mortality in the critically ill [25]. Discrimination of sepsis from noninfectious etiologies of SIRS is difficult because both conditions display identical clinical symptoms [26, 27]. Timely discrimination of sepsis from SIRS is essential for initiation of early goal directed therapy. To date there is no reliable biomarker available, which facilitates this diagnosis. In a recent study using mass spectrometry we identified CAAP48 as potential discriminatory sepsis biomarker [28]. Further analysis of AAT and its fragments revealed an increased proteolytic activity in patients with severe sepsis resulting in high peak intensities of AAT-fragments compared to severe SIRS of noninfectious origin.

In the current study we analyzed the biological function of CAAP48 and CAAP47 as well as several control peptides on human polymorphonuclear neutrophils (PMN), in order to explore effects of the peptides on PMN that might elucidate our clinical findings [28]. We also analyzed the peptide concentrations in severe SIRS and severe sepsis patients as well as HIV infected patients to apply CAAP48 as a sepsis biomarker.

## 2. Material and Methods

### 2.1. Patients

Four different patient groups were enrolled in the study (severe SIRS following cardiac surgery (*n* = 19), severe SIRS following traumatic injury (*n* = 17), severe sepsis (*n* = 19), and HIV infected patients (*n* = 23)). For validation purposes, samples from patients with severe SIRS following cardiac surgery as well as with severe sepsis comprised patients from our previous qualitative study [28]. Patients with severe SIRS following traumatic injury (*n* = 17) were enrolled with an injury severity score (ISS) ≥16, prehospital systolic blood pressure <90 mmHg or metabolic acidosis, and the condition that time point of trauma being not longer than 12 hours ago. Nine, six, and eight HIV patients were enrolled with virus titers of <100, 100–10000, and >10000 cop/mL, respectively. Healthy volunteers were included for AAT genotype and phenotype correlation and for the cell experiments. Patients less than 18 years old, pregnant or lacking informed consent, were excluded. The study and all protocols were approved by the local ethics committee (0713-08/01, 3309-11/11, 2734-12/09).

### 2.2. Patient Characteristics

Severe SIRS patients after cardiac surgery or polytrauma do not differ in their clinical characteristics compared to patients with severe sepsis as demonstrated, for example, by their SOFA score (Table 1).

### 2.3. Peptides

Peptides were synthesized by solid phase peptide synthesis and purified by HPLC with greater 95% purity (Prof. D. Imhof, Pharmaceutical Institute, Pharmaceutical Chemistry I, University of Bonn, Germany). Shortly, peptides were synthesized automatically by a standard Fmoc [*N*-(9-fluorenyl)methoxycarbonyl] protocol by manual SPPS or on an EPS221 peptide synthesizer (Intavis Bioanalytical Instruments AG, Cologne, Germany). In general, coupling reactions were performed using Fmoc-amino acids (5 equiv) activated with HBTU (5 equiv) in the presence of* N*-methylmorpholine/DMF (1 : 1) for 5–15 min (double couplings). Fmoc removal was effected by treating the resin twice with piperidine in DMF (20%). All deprotection and coupling steps were followed by intensive washings with DMF and dichloromethane (CH_2_Cl_2_), alternately. Peptide cleavage and deprotection were accomplished with reagent K (trifluoroacetic acid (TFA)/water/phenol/thioanisole/ethanedithiol 82.5 : 5 : 5 : 5 : 2.5) for 4 hours at room temperature. The crude peptides were precipitated with diethyl ether, centrifuged, and washed several times with diethyl ether. The yields of the crude peptides varied between 60 and 70%. The crude peptides were purified by semipreparative reversed-phase HPLC with a Shimadzu LC-8A system equipped with a C18 column (Knauer Eurospher 100, Berlin, Germany). The gradient elution system was 0.1% TFA in water (eluent A) and 0.1% TFA in acetonitrile/water (9 : 1, eluent B). The peptides were eluted with a gradient of 0–50% eluent B in 120 min and a flow rate of 10 mL/min. The peaks were detected at 220 nm. Collected fractions were combined, freeze-dried, and stored at −20°C. Purities of the peptides were confirmed by analytical reversed-phase HPLC with a Shimadzu LC-10AT chromatograph (Duisburg, Germany) equipped with a Vydac 218TP54 column (C18, 5 *μ*m particle size, 300 Å pore size, 4.6 × 25 mm). The peptides were analyzed by gradient elution at a flow rate of 1 mL/min, where eluent A was 0.1% TFA in water, and eluent B was 0.1% TFA in acetonitrile; detection was at 220 nm.

Table 2 gives an overview of the different peptides employed.

### 2.4. Peptide Quantification

We developed a LC-MS/MS method for quantification of CAAP48 and the SNP-variant CAAP47 and determined its concentration in the critically ill. Because of divergent characteristics of synthetic and endogenous CAAP48, two plasma samples with defined CAAP48 concentrations were used as calibrants for quantification by two-point calibration. This LC-MS-MS method was validated according to the FDA “Guidance for Industry Bioanalytical Method Validation.”

Plasma was diluted 1 : 10 in 20 mM Tris + 1% formic acid (FA) and incubated for 5 hours at room temperature. After incubation samples were transferred to LC-MS vials and kept at 4°C in the tray. Samples were analyzed on a Liquid Chromatography system LC20 AVP (Shimadzu, Japan) connected to an API3000*™* mass spectrometer (AbSciex, USA). 5 *μ*L of diluted sample was injected onto a Kromasil-Eternity-2.5-C18 2.1 × 100 mm UHPLC column (Dichrom GmbH, Marl, Germany) with a precoupled KrudKatcher Ultra HPLC In-Line Filter 0.5 *μ*m (Phenomenex, USA). The peptide was eluted with solvent A (H_2_O + 0.1% FA) and solvent B (AcN + 0.1% FA), starting at 90% A for 2 min, followed by a 6 min linear gradient to 40% A, 5 min 40% A, 1 min linear gradient to 90% A, and 6 min 90% A with a flow rate of 200 *μ*L/min. The column oven was set at 38°C. Two peptides were chosen for fragmentation: the 5-fold charged CAAP48 peptide (4789.7 Da) at 958.9 Da and the 6-fold charged CAAP48 peptide at 799.3 Da. The following transitions could be detected at high intensities: 799.3/873.6, 799.3/243.4, 799.3/314.3, 958.9/1091.8. MS parameters were CXP: 27, DP: 60, EP: 10, CAD: 12, CUR: 22, GS1: 40:, GS2: 50, IS: 5500, TEM: 600. CE was optimized for the single fragments (874.4: 27, 1091.5: 33, 243.4: 39, and 314.3: 28). For detection of the CAAP47 peptide (4775.7 Da) equivalent transitions were chosen at 797.0/873.6, 797.0/243.4, 956.167/1088.4, and 797.0/314.3.

Calibrants were created by serially diluting synthesized CAAP48 peptide and CAAP47 peptide in 40 g/L albumin solution (20% Albunorm Human Albumin, Octapharma, Langenfeld, Germany, diluted in H_2_O_bd_). Samples were measured in duplicate.

### 2.5. Method Validation

Method validation was carried out on the basis of the FDA “Guidance for Industry Bioanalytical Method Validation.” Shortly, linearity was determined measuring 6 consecutively diluted standards 10 times and generating a linear standard curve with the mean values. Limit of detection (LOD) was determined to be the mean of 10 blank values plus 2 standard deviations (SD). The lower limit of quantification (LLOQ) was determined to be the lowest concentration that could be measured with acceptable precision (CV < 20%). Intra- and interday precision and accuracy were determined measuring three samples at 6.4, 11.4, and 36 ng/*μ*L 8 times a day for 10 consecutive days, respectively. Mass spectrometric selectivity is defined as a constant ratio of quantifier transition (243.4) to qualifier transition (1091). Recovery was determined by spiking three standard concentrations in EDTA plasma of donor plasma. Autosampler stability was determined by measuring three samples 8 times in a time range of 24 hours. Freeze/thaw-stability was determined by thawing and freezing two samples on 8 consecutive days. Parallelism was determined by diluting a sample consecutively in buffer and referring the concentrations of the dilutions to the original concentration.

Validation resulted in a linear calibration curve in a range of 2–200 ng/*μ*L with a coefficient of determination of 1. The LOD was 2.03 ng/*μ*L; the LLOQ was about 6 ng/*μ*L (interday precision = 15% at 6.4 ng/*μ*L). Intraday accuracy was determined to be 0.98, 1.13, and 0.88 for the three respective concentrations. Interday accuracy was determined to be 1.07, 1.01, and 0.95. Intraday precision was 4, 11, and 11%; interday precision was 15, 13, and 10%. Specificity, defined as the transition ratio of 243/1091, had a CV of 15%. Autosampler stability for 24 hours was given with CVs of 13, 11, and 11%. Two samples at 7 and 47 ng/*μ*L were thawed and refrozen on eight consecutive days, without relevant impact on peptide concentration (CV 9 and 5%). Mean recovery of 1, 5, and 10 ng CAAP48 peptide was 87.4, 70.5, and 89.6%, respectively. Parallelism was defined as the deviation for two 1 : 2 dilutions of the 36 ng/*μ*L sample, which was 4.91 and 7.32%.

### 2.6. Isolation of Human Polymorphonuclear Neutrophils

Human peripheral blood was collected in EDTA-tubes from healthy volunteers after informed consent. In accordance with Helsinki Declaration, the design of this study and the enforcement of the experiments were exhaustively explained to the participants, and written informed consent was obtained. Human PMN were isolated from the peripheral blood using Polymorph-Prep (PROGEN Biotechnik) as specified by Wozniok et al. (2008) [30]. Purified PMN were resuspended in RPMI 1640 containing 5% heat inactivated human serum. The purity of PMN was ≥90%.

### 2.7. Culture Conditions

The lyophilized peptides were dissolved in sterile 1x PBS w/o Ca and Mg at a concentration of 400 *μ*M (1.92 *μ*g/*μ*L) and stored at −20°C. For each experiment fresh aliquots were thawed. The synthetic peptides were used at assay concentrations between 5 *μ*M (24 ng/*μ*L) and 100 *μ*M (480 ng/*μ*L). In each functional assay 1 ng/mL phorbol myristate acetate (PMA) was used as positive control and 1x PBS diluted in RPMI 1640 (1 : 2) as negative control to show that PBS has no influence on PMN. Isolated PMN were incubated with different concentrations (5 *μ*M (24 ng/*μ*L); 20 *μ*M (96 ng/*μ*L); 40 *μ*M (192 ng/*μ*L); and 100 *μ*M (480 ng/*μ*L)) of the synthetic peptides for 30 min at 37°C on a roll-shaker.

### 2.8. Fluorescence-Activated Cell Sorting Analysis (FACS)

Analyses of the expression of cell surface activation markers were performed using differential FACS staining. PMN were labeled with CD66b-FITC (clone 80H3, AbD Serotec, Puchheim, Germany), CD62L-APC (clone DREG-56, BioLegend, Fell, Germany), CD63-V450 (clone H5C6, BD Biosciences, Heidelberg, Germany), and CD69-PE (clone L78, BD Biosciences, Heidelberg, Germany) at 4°C for 30 min, washed with CellWash (BD Biosciences, Heidelberg, Germany), and centrifuged at 300 g for 5 min. Cells were analyzed using a FACS Canto II flow cytometer (BD Biosciences, Heidelberg, Germany). The software FlowJo 7.6.4 (BD Biosciences, Heidelberg, Germany) was used for analysis.

### 2.9. Oxidative Burst

The PMN oxidative burst was measured using a commercially available test kit (Orpegen Pharma, Heidelberg, Germany), according to the manufacturer's recommendations. Results were expressed as median fluorescence intensity of the whole PMN population.

### 2.10. Annexin V-FITC/Propidium Iodide Apoptosis Assay

Viability of cells was quantified using BD Pharmingen*™* Annexin V: FITC Apoptosis Detection Kit I (BD Biosciences, Heidelberg, Germany). In a calcium-enriched binding buffer (1x) 1 × 10^6^ cells/mL were double stained with Annexin V-FITC and propidium iodide (PI) for 15 min at RT in the dark. After incubation the cells were washed with 1x binding buffer and analyzed by flow cytometry.

### 2.11. Quantification of Secreted Proteins

PMN were coincubated with 10–100 *μ*M synthetic peptide for 30 min, 1 h, 2 h, and 4 h. Cytokine concentrations within the supernatant of coincubated PMN were determined by Bio-Plex assay (Pro Human Cytokine 14-plex Assay, BioRad Laboratories GmbH, München, Germany), according to the manufacturer's instructions. Supernatants were stored at −80°C until analysis.

### 2.12. PMN Migration/Chemotaxis Assay

Isolated PMN were resuspended in RPMI 1640 with added heat inactivated human serum (5%) at the concentration of 1 × 10^6^ cells/mL. Cell migration/chemotaxis was measured by using 6.5 mm Transwell® with 3.0 *μ*m pore polyester membrane insert (Corning GmbH HQ, Wiesbaden, Germany). The effects of CAAP48 and the SNP-variant CAAP47 were tested on spontaneous migration. The PMN suspension was added to the upper surface of the membrane and RPMI 1640 medium alone and CAAP48 or CAAP47 to the lower compartment. CAAP48 and CAAP47 were used at a concentration of 100 *μ*M (480 ng/*μ*L). The number of cells migrating to the lower wells of the chamber was assessed by microscopy and using a hematological analyzer (BC-5300, Mindray Medical Germany GmbH, Darmstadt) after 60 min.

### 2.13. Statistics and Data Analysis

Peak areas, calibration curves, and concentrations were calculated using Analyst 1.51 (AbSciex, USA). Statistical analysis including testing for significance and ROC-curve analysis was carried out with SPSS 19 (IBM, USA). Normal distribution of data was checked for each patient group. If data showed normal distribution the parametric Student *t*-test for two independent samples was used; otherwise the nonparametric Mann-Whitney  *U*-test for two independent samples was applied for determination of statistical significance. Cutoff values from ROC-curves were determined using the Youden index [31]. Determination of significant differences between the areas under two independent ROC-curves was performed according to Hanley and McNeil [32]. For the cell experiments three to five independent replicates using cells from nonidentical donors were analyzed. The level of significance was 0.05.

## 3. Results

### 3.1. Activation of Neutrophils by CAAP48

**Figure 1:**
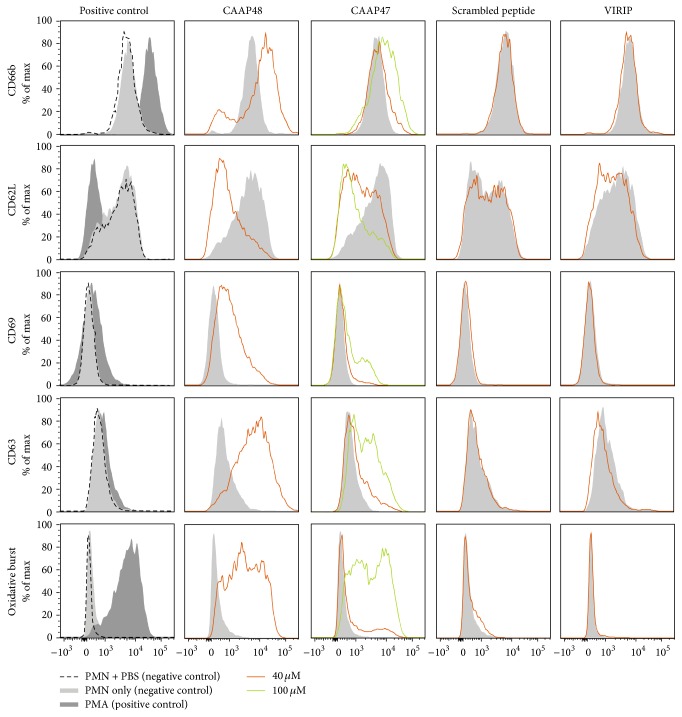
Incubation of neutrophil granulocytes (PMN) with CAAP48 results in cellular activation. PMN activation is shown 30 min after incubation with CAAP48 and the other AAT peptides by changes in the surface expression levels of activation/degranulation markers CD66b, CD63, CD69, and CD62L. Oxidative Burst was determined by detection of intracellular generated reactive oxygen species (oxidation of dihydrorhodamine-123 to rhodamine-123, R-123). Data obtained by using 40 *µ*M and 100 *µ*M peptide concentrations of one exemplary experiment out of four independent experiments are shown. Phorbol myristate acetate (PMA) was used as positive control and 1x PBS diluted in RPMI 1640 (1 : 2) as well as PMN in RPMI 1640 without PBS served as negative control.

To investigate the pathophysiological function of CAAP48 we assessed PMN activation in response to CAAP48. As activation markers we used CD66b, CD63, and CD69, which are upregulated in activated PMN as well as CD62L, which is constitutively highly expressed on PMN and rapidly decreases upon activation by enzymatic shedding. PMN were highly activated by 40 *μ*M (192 ng/*μ*L) CAAP48 as demonstrated by increased expression (fold change of median fluorescence intensity ± SD of four independent experiments, compared with control) of CD66b (2.11 ± 0.77), CD63 (3.05 ± 0.53), and CD69 (1.62 ± 0.19) and decreased expression of CD62L (0.1 ± 0.07). Differential regulation of surface expression of respective CD's from one representative experiment is shown in Figure 1. Activation of PMN by CAAP48 was concentration-dependent, while CAAP47 showed only a slight activation at 100 *μ*M (480 ng/*μ*L) (CD66b: 1.14 ± 0.03, CD63: 1.12 ± 0.08, CD69: 1.12 ± 0.14, and CD62L: 0.67 ± 0.19). Furthermore we observed no effect on PMN activation after incubation with VIRIP (CD66b: 1.0 ± 0.04, CD63: 0.9 ± 0.15, CD69: 0.89 ± 0.06, and CD62L: 0.85 ± 0.07) and scrambled peptide (CD66b: 1.05 ± 0.04, CD63: 1.0 ± 0.04, CD69: 1.0 ± 0.06, and CD62L: 0.9 ± 0.14). Analysis of PMN activation by measurement of oxidative burst in the presence of CAAP48, CAAP47, VIRIP, and scrambled peptide showed a dose-dependent induction of oxidative burst by CAAP48. In contrast, neither VIRIP nor the scrambled peptide had any effect while CAAP47 induced oxidative burst only at 100 *μ*M (480 ng/*μ*L) (Figure 1).

### 3.2. CAAP48 Function Is Mediated by Its Hairpin Structure

**Figure 2:**
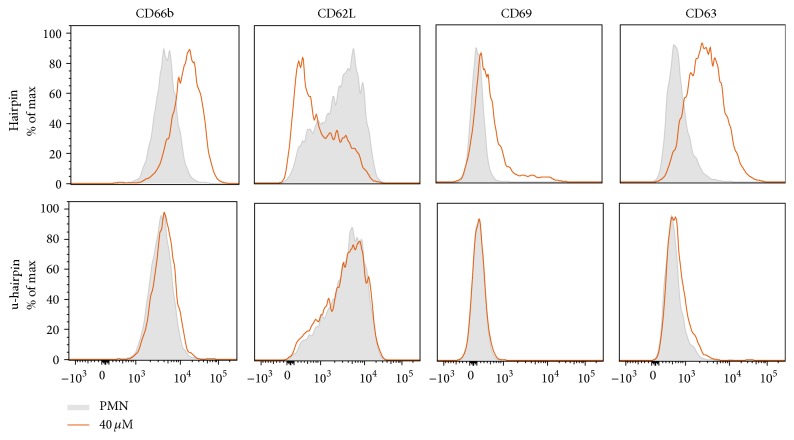
The hairpin structure of CAAP48 is responsible for the function of the peptide. For identification of the functional domain of the peptide, we incubated fresh isolated PMN with the hairpin peptide and the sequence until the hairpin (u-hairpin) for 30 min. Cells were analyzed for surface expression of CD66b, CD63, CD69, and CD62L by flow cytometry. The hairpin peptide activates PMN, while the u-hairpin peptide shows no activation. Differential expression of activation markers is shown in histograms of one representative experiment out of four independent experiments using peptide concentrations of 40 *µ*M.

CAAP48 contains a hairpin region and a linear sequence [33]. In order to identify the functional domain of the peptide, we incubated PMN with a peptide that comprises the hairpin structure (designated as hairpin peptide, corresponding to AAT residues 369–394) and another peptide, which represents the sequence until the hairpin (designated as u-hairpin peptide, corresponding to AAT residues 353–368). A concentration-dependent PMN activation was only observed for the peptide containing the hairpin structure (CD66b: 2.55 ± 0.42, CD63: 3.42 ± 1.59, CD69: 2.0 ± 0.5, and CD62L: 0.52 ± 0.08 after treatment with 40 *μ*M (192 ng/*μ*L) hairpin peptide, compared to control) (Figure 2).

### 3.3. CAAP47/48 Affects Neutrophil Viability

**Figure 3:**
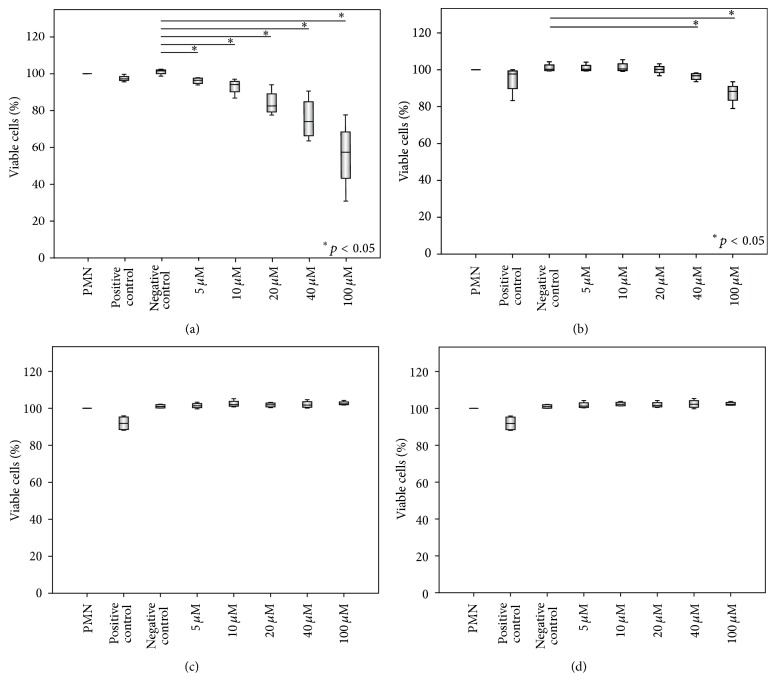
Neutrophil viability in response to CAAP48, CAAP47, scrambled peptide, and VIRIP. After 30 min peptide incubation, PMN (1 × 10^6^ cells/mL) were double stained with Annexin V-FITC and propidium iodide (PI) for 15 min and analyzed by flow cytometry. The percentage of viable cells (PI and Annexin negative) is illustrated for different peptide concentrations. CAAP48 (a) leads to a marked reduction of the viability of neutrophils already at 5 *μ*M (24 ng/*μ*L) while CAAP47 (b) influences viability of PMN not before 40 *μ*M (192 ng/*μ*L). Both the scrambled peptide (c) and VIRIP (d) had no effect. Data of four independent experiments are shown. PMA was used as positive control and 1x PBS diluted in RPMI 1640 (1 : 2) as well as PMN in RPMI 1640 without PBS served as negative control.

We hypothesize that CAAP48 can modulate neutrophil function by inducing apoptosis. To test this possibility, purified neutrophils were incubated with CAAP48* in vitro* and stained with Annexin V and PI after 30 min. We found that the percentage of viable neutrophils (Annexin V− PI−) was significantly reduced after treatment with CAAP48 in a dose-dependent manner. The influence of CAAP47 on the viability of PMN was significantly smaller. By contrast, both VIRIP and the scrambled peptide showed no effect on the viability of PMN (Figure 3).

### 3.4. Chemotactic Activity of CAAP48 and the SNP-Variant CAAP47

**Figure 4:**
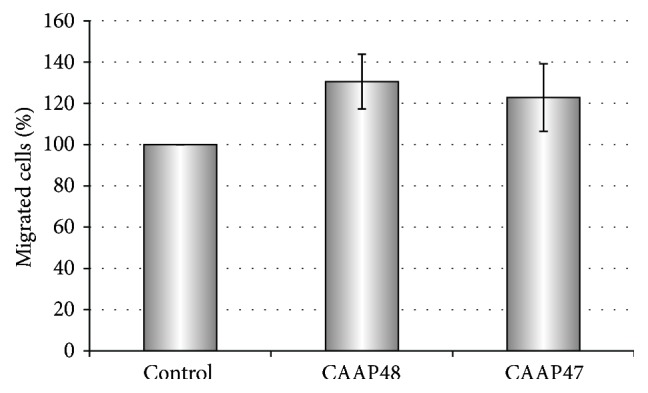
Chemotactic response of neutrophils to CAAP48 and the SNP-variant CAAP47. The chemotactic response of isolated neutrophils to CAAP48 and the SNP-variant CAAP47 was examined using disposable Boyden chambers. The number of cells migrating to the lower wells of the chamber was assessed by microscopy and using a hematological analyzer after 60 min. Control: RPMI 1640 medium alone; CAAP48 and CAAP47 were used at a concentration of 100 *μ*M (480 ng/*μ*L). CAAP48/CAAP47 dependent migration was normalized to spontaneous migration in controls. Each bar represents the mean ± SD of three independent experiments.

The chemotactic response of isolated neutrophils to CAAP48 and the SNP-variant CAAP47 was examined using disposable Boyden chambers. After 60 min, 100 *μ*M CAAP48 as well as CAAP47 lead to significant migration of PMN compared to spontaneous migration in controls without stimulus. As shown in Figure 4 migration could be induced by CAAP48 and CAAP47 up to 130 ± 13% (SD) and 122 ± 16%, respectively (*p* < 0.05). Migration experiments performed at 40 *μ*M peptide concentration did not result in increased migration compared to controls without stimulus (data not shown).

### 3.5. Cytokine Release of PMN Triggered by CAAP48 and CAAP47

**Figure 5:**
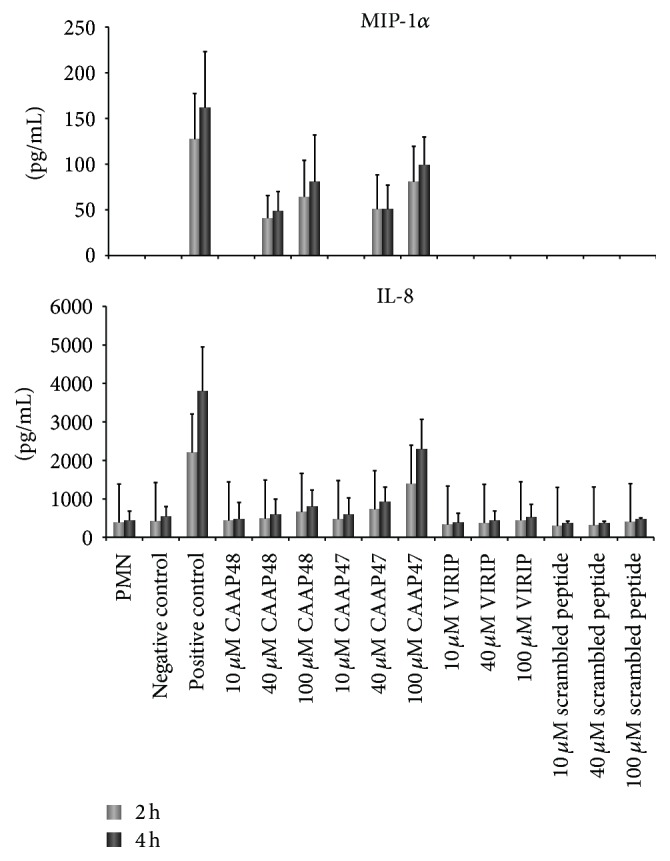
MIP-1*α* and IL-8 concentrations after incubation of PMN with AAT peptides. The concentration of MIP-1*α* and IL-8 in the cell culture supernatant of PMN after peptide stimulation is shown. PMN were incubated with different AAT peptides for two and four hours at concentrations as indicated. MIP-1*α* and IL-8 were quantified by Bio-Plex. Each bar represents the mean ± SD of three independent experiments. The lower limit of detection for MIP-1*α* measurements is 1.6 pg/mL and 1.0 pg/mL for IL-8. MIP-1*α* and IL-8 are released in a dose-dependent manner upon PMN incubation with CAAP47 and CAAP48, while no dose-dependent MIP-1*α* or IL-8 release could be observed upon stimulation with VIRIP or the scrambled peptide compared to the negative control (1x PBS diluted in RPMI 1640 (1 : 2)). PMA stimulation served as positive control.

To analyze whether neutrophil activation by CAAP47 and CAAP48 is accompanied by release of cytokines, 14 different cytokines (IL-1*β*, IL-2, IL-4, IL-6, IL-8, IL-10, IL-12, G-CSF, GM-CSF, INF-*γ*, MCP-1, MIP-1*α*, TNF*α*, and VEGF) were quantified after incubation of PMN with CAAP48 or CAAP47 at different time points. In contrast to PMN activation, which could be demonstrated already after 30 min for example, by increased surface expression of CD66b, CD63, and CD69, a dose-dependent increase of cytokines, compared to the negative control, was observed for the chemokines MIP-1*α* and IL-8 after 2 and 4 hours of incubation with CAAP48 and CAAP47 (Figure 5).

### 3.6. CAAP47/48 Concentrations in Different Patient Groups

**Figure 6:**
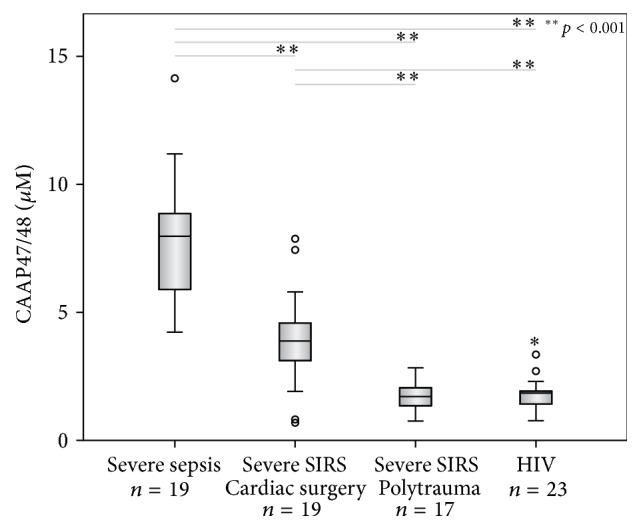
Boxplots of CAAP47/48 concentrations. Boxplots of CAAP48 concentrations in severe sepsis and SIRS patients following cardiac surgery and polytrauma are shown. CAAP48 concentrations comprise CAAP48 or CAAP47 concentrations for homozygous individuals and CAAP47/48 concentrations for heterozygous individuals. Circles represent mild outliers and asterisks extreme outliers. *p* values for significant differences between groups are indicated with respective lines. Severe sepsis patients exhibit significant higher CAAP47/48 concentrations compared to the severe SIRS and HIV infected patients.

**Figure 7:**
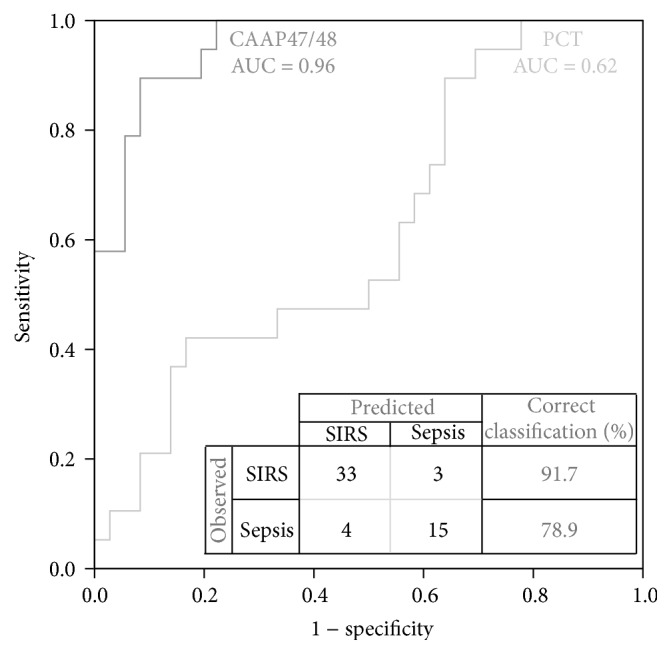
Receiver operating characteristic (ROC)-curve analysis comparing the test-characteristics of CAAP47/48 and procalcitonin (PCT) for discrimination of patients with severe sepsis (*n* = 19) and patients with severe SIRS (polytrauma and cardiac surgery, *n* = 36). The area under the ROC-curve (AUROC) for CAAP47/48 (0.96) is significantly higher than for PCT (0.62) demonstrating a greater diagnostic accuracy of CAAP47/48 compared to PCT. The optimal cutoff, which is defined to be the concentration at which patients are classified into the SIRS or sepsis group with highest sensitivity and specificity, is 4.9 *μ*M. Severe SIRS and severe sepsis patients can be differentiated with 91.7% specificity and 78.9% sensitivity.

Concentrations of CAAP47/48 determined in samples from the respective patient groups are shown in Figure 6. CAAP47/48 is defined to be the resulting C-terminal peptide after Phe^352^-Leu^353^ cleavage of AAT (42 amino acids). CAAP47/48 concentrations were significantly higher in severe sepsis patients compared to severe SIRS patients following cardiac surgery (*p* < 0.001) or polytrauma (*p* < 0.001). The median concentration in severe sepsis was 7.97 *μ*M, which is a 2–4-fold increase compared to the SIRS groups. ROC-curve analysis of CAAP47/48 concentration for discrimination of severe sepsis and severe SIRS due to cardiac surgery and polytrauma (AUROC = 0.96) demonstrated a significantly higher diagnostic accuracy for the discrimination of severe SIRS and severe sepsis, compared to the conventional used sepsis marker procalcitonin (PCT, AUROC = 0.62, *p* < 0.05) (Figure 7). Only four out of 19 severe sepsis and three out of 36 severe SIRS cases were misclassified, yielding a sensitivity of 78.9% and a specificity of 91.7%.

It has been stated that a peptide encompassing amino acids 1–20 of CAAP48, designated VIRIP, is expressed in HIV infected patients and correlates with viremia [18, 23]. This suggests that CAAP48 might also be elevated in HIV patients. However, CAAP48 concentrations did not correlate with virus titer (*r* = 0.1, data not shown) and were significantly lower compared to severe sepsis patients (*p* < 0.001) and severe SIRS patients due to cardiac surgery (*p* < 0.001).

## 4. Discussion

Recently, we have shown that mass spectrometry-based protein patterns may be helpful in the diagnosis of severe sepsis versus noninfectious systemic inflammatory response syndrome. We analyzed protein expression patterns from plasma using SELDI-TOF-mass spectrometry and identified a cleaved peptide of AAT (CAAP48) as potential sepsis biomarker [28]. Quantification of CAAP47/48 further substantiates our previous findings that generation of CAAP47/48 is highly specific for sepsis compared to systemic inflammation of noninfectious etiology_,_ whereby a higher concentration of the peptide indicates an underlying infection. Compared to patients with systemic inflammation, CAAP47/48 concentrations are neither elevated in HIV infected patients nor did they correlate with virus titers, leading to the assumption that CAPP47/48 might be indicative for bacterial infections. This is of interest because CAAP48 comprises the sequence of the so-called peptide VIRIP (amino acids 1–20 of CAAP48), which has been shown to be elevated in HIV infected individuals where it acts as inhibitor of HIV entry in T-cells [18, 23].

**Figure 8:**
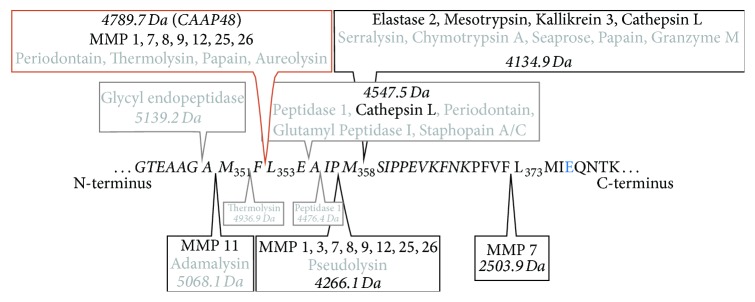
Cleavage sites in the AAT reactive center loop (RCL). Shown are the known cleavage sites in the RCL (italic) of AAT with respective enzymes gathered from the MEROPS database [29]. Human proteases are displayed in black and proteases of other species in grey. Also for every cleavage site the respective molecular weight of the C-terminal peptide is indicated. Cleavage between Phe^352^ and Leu^353^ leads to the generation of CAAP48 (red frame). However in the presence of SNP rs1303 cleavage at this site leads to the generation of CAAP47, a peptide with an E > D substitution at the position indicated in blue.

There is increasing evidence that proteases, capable of generating CAAP47/48, play an important role in the course of bacterial infections. Thus, all proteases that cut AAT in the reactive center loop were gathered from the MEROPS peptide database [29], as illustrated in Figure 8. Of note, all known human proteases that might liberate CAAP47/48 (red frame in Figure 8) from AAT belong to the group of matrix metalloproteases (MMPs), which have been shown to be elevated in sepsis [34–36]. From the listed proteases the bacterial proteases (periodontain, aureolysin, and thermolysin) and the human matrilysins (MMP7, MMP26) are of special interest, since they have been shown to efficiently cut AAT at Phe^352^-Leu^353^, therefore contributing to the generation and amplification of CAAP47/48 in the context of sepsis [12, 37–43]. Also, MMP-7, expressed in mononuclear phagocytes and many exocrine and mucosal tissues, shows a strong and sustained induction after exposure of mucosal epithelial tissue to microorganisms, such as* P. aeruginosa* and* E. coli *[44].

There is disagreement if CAAP47/48 is released from cleaved AAT. It has been shown that C-terminal AAT-fragments are captured noncovalently within the cleaved AAT-complex under native conditions [11, 12, 33]. However AAT-fragments have also been isolated from human blood and tissues and even revealed pathophysiological functions [13–23]. Furthermore, by* in vitro* cleavage of AAT with thermolysin and subsequent anion exchange chromatography, we found a fraction of free CAAP48 upon cleavage, while the main part remains within the AAT-complex (data not shown). Since we assume that CAAP47/48, captured noncovalently within the cleaved AAT-complex, will be liberated during the sample preparation step described here, quantification of CAAP47/48 by LC-MS/MS will result in measurement of total CAAP47/48 comprising the free and the bound fraction.

We could demonstrate* in vitro* neutrophil activation and oxidative burst at comparable CAAP48 concentrations as determined in sepsis patients, while the SNP-variant CAAP47 was less effective and the scrambled control peptide had no effect at pathophysiological relevant concentrations. These* in vitro* findings are in concordance with previous results of another C-terminal fragment of AAT (C-36 peptide, corresponding to AAT amino acid sequence 358–396) showing significantly increased neutrophil degranulation and superoxide generation [22]. This indicates that the proteolytic cleavage of AAT at sites of inflammation may inverse the anti-inflammatory effect of AAT and contribute to neutrophil recruitment and activation, leading to an aggravation of the inflammation.

Human PMN play an important role in the immune defense against bacterial and fungal infections. However, inappropriate or dysregulated activation of PMN may also lead to persisting tissue damage, which results in the generation of DAMPs, contributing to the pathogenesis of SIRS. The intensity of an inflammatory response and the degree of PMN-mediated tissue injury are affected by apoptosis of PMN [45, 46]. It is well known that neutrophil apoptosis is delayed in sepsis [47–49], most likely due to certain cytokines. In contrast, we could show that CAAP48 accelerates cell death in PMN.

Moreover, activated PMN have been shown to synthesize and secrete cytokines and chemokines in response to a number of stimuli, which can subsequently activate both neutrophils and other immune cells. We determined the concentrations of 14 different cytokines and could show that the release of MIP-1*α*, a chemokine which recruits target cells to sites of inflammation, was influenced by CAAP48 and the SNP-variant CAAP47. In summary, these results highlight the central function of AAT in the regulation of neutrophils, independent of its antiprotease activity. It has also been shown that cleaved fragments of AAT act as potent neutrophil chemoattractants [15, 22]. We could verify these results and show that neutrophil migration could be increased by CAAP48 and CAAP47, again suggesting that proteolytically cleaved AAT represents a powerful proinflammatory molecule [22].

**Figure 9:**
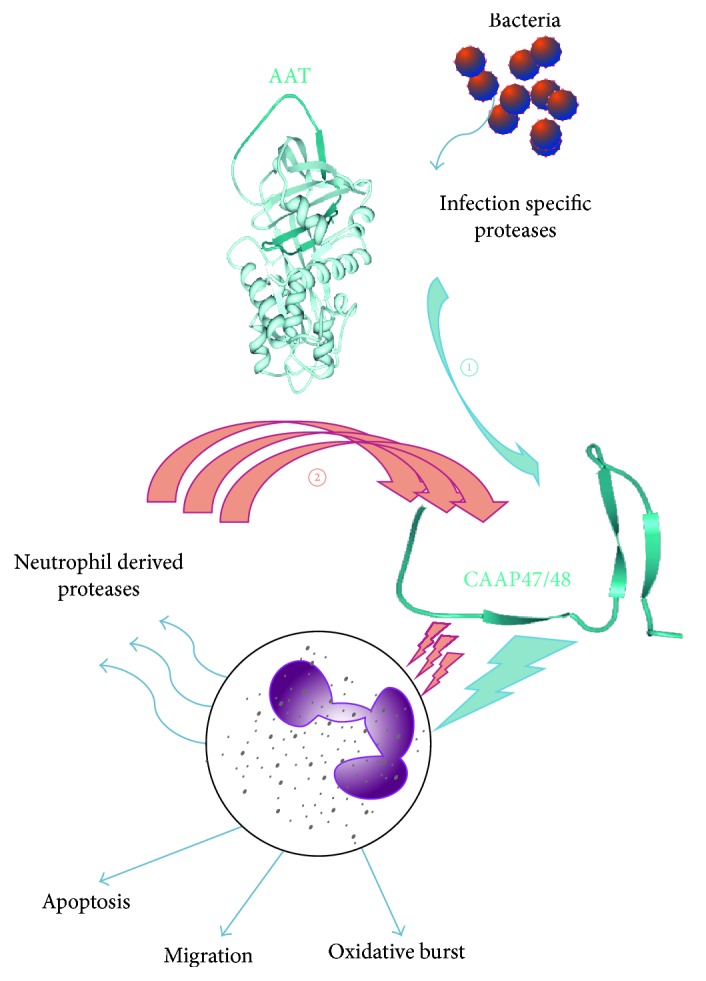
Hypothetical pathway illustrating CAAP47/48 generation by infection specific proteases. (1) In severe sepsis infection specific proteases are activated that lead to AAT cleavage and thus generation of CAAP47/48 (blue arrows). This proteolytic fragment activates PMN, which in turn leads to secretion of further proteases from PMN that will result in additional cleavage of ATT and accumulation of CAAP47/48 (2), that subsequently activates other PMN (red arrows). Due to the activation of further PMN and the induction of migration, cell death, and oxidative burst, this activation loop contributes to a strong enhancement of inflammation.

In summary, these findings support our hypothesis that in severe sepsis infection specific proteases are activated that lead to cleavage of AAT between Phe^352^ and Leu^353^ and are responsible for CAAP47/48 generation. This proteolytic peptide vastly activates PMN, which in turn secrete neutrophil-derived proteases that lead to generation of additional CAAP47/48 molecules for subsequent activation of further PMN (Figure 9).

Besides its importance as sepsis biomarker this study emphasizes the immunomodulatory functions of CAAP47/48 in the pathophysiology of sepsis. However, the important role of CAAP47/48 as sepsis biomarker has to be further evaluated in prospective collected patient cohorts with defined bacterial infections, different foci of infection, and in the time course of sepsis to clarify and validate the role of CAAP47/48 in early identification of patients at risk and timely initiation of appropriate antibiotic treatment. Studies are under way for development of a robust and highly sensitive method that will allow quantification of the free fraction of CAAP47/48 in the clinical routine setting.

## Figures and Tables

**Table 1:** Patient characteristics.

	Sepsis	SIRS cardiac surgery	SIRS traumatic injury	HIV
*n*	19	19	17	23
Age ([years], mean ± SD)	63.05 ± 15.29	68.89 ± 7.43	39.29 ± 14	39 ± 14.97
Gender (m/f)	11/8	11/8	12/5	17/6
SOFA^*∗*^ (mean ± SD)	11.42 ± 3.55	10.21 ± 3.97	10.76 ± 2.44	—
Procalcitonin ([ng/mL], median (range))	5.34 (163.9)	7.28 (159.24)	0.49 (20.2)	0.07 * *(0.24)^*∗∗*^
Mechanical ventilation (%)	84.2	68.4	94.1	—
MDI/CDI	13/6	0/0	0/0	23/0
Pathogen (gram^+^/gram^−^/fungal/mixed infection/viral)	4/7/1/1/0	0/0/0/0/0	0/0/0/0/0	0/0/0/0/23
*HIV titer (<100/100–10000/>10000)*	—	—	—	9/6/8
*Staphylococcus aureus*	2	—	—	—
*Escherichia coli*	4	—	—	—
*Enterobacter*	1	—	—	—
*Klebsiella*	2	—	—	—
*Candida* (other than *albicans*)	1	—	—	—
*Enterococcus*	3	—	—	—

^*∗*^SOFA score: Sequential Organ Failure Assessment Score. Subscores on SOFA range from 0 to 4 for each of six organ systems, with an aggregate score of 0 to 24 and with higher scores indicating more severe organ dysfunction.

^*∗∗*^Procalcitonin values were determined in 18 out of 23 HIV patients, MDI: microbiologically documented infection, CDI: clinically suspected infection.

**Table 2:** AAT peptides.

Peptide	Corresponds to AAT residues	Average molecular weight [Da]	Sequence
CAAP48	353–394	**4**7**8**9	H-LEAIPMSIPPEVKFNKPFVFLMI**E**QNTKSPLFMGKVVNPTQK-OH
CAAP47 (E > D substitution)	353–394	**4**7**7**5	H-LEAIPMSIPPEVKFNKPFVFLMI**D**QNTKSPLFMGKVVNPTQK-OH
Hairpin peptide	369–394	2980	H-PFVFLMIEQNTKSPLFMGKVVNPTQK-OH
u-hairpin	353–368	1813	H-LEAIPMSIPPEVKFNK-OH
VIRIP	353–372	2303	H-LEAIPMSIPPEVKFNKPFVF-OH
Scrambled peptide		4789	H-KKFISPFVKMPNFVTSGPVIPQNEKFLMEQPKLMVPITLENA-OH

CAAP47 and CAAP48 were named according to the first and third number (bold) of their respective molecular weight.
